# Analysis of two sequential SARS-CoV-2 outbreaks on a haematology-oncology ward and the role of infection prevention

**DOI:** 10.1016/j.infpip.2023.100335

**Published:** 2024-01-06

**Authors:** W.C. van der Zwet, E.A. Klomp-Berens, A.M.P. Demandt, J. Dingemans, B.M.J.W. van der Veer, L.B. van Alphen, J.A.M.C. Dirks, P.H.M. Savelkoul

**Affiliations:** a Department of Medical Microbiology, Infectious Diseases & Infection Prevention, Care and Public Health Research Institute (CAPHRI), Maastricht University Medical Center, Maastricht, The Netherlands; b Division of Hematology, Department of Internal Medicine, GROW School for Oncology and Developmental Biology, Maastricht University Medical Center, Maastricht, The Netherlands

**Keywords:** Infection control, SARS-CoV2, Whole Genome Sequencing, Outbreak, Screening asymptomatic patients, Screening asymptomatic healthcare workers

## Abstract

Two SARS-CoV-2 nosocomial outbreaks occurred on the haematology ward of our hospital. Patients on the ward were at high risk for severe infection because of their immunocompromised status. Whole Genome Sequencing proved transmission of a particular SARS-CoV-2 variant in each outbreak. The first outbreak (20 patients/31 healthcare workers (HCW)) occurred in November 2020 and was caused by a variant belonging to lineage B.1.221. At that time, there were still uncertainties on mode of transmission of SARS-CoV-2, and vaccines nor therapy were available. Despite HCW wearing II-R masks in all patient contacts and FFP-2 masks during aerosol generating procedures (AGP), the outbreak continued. Therefore, extra measures were introduced. Firstly, regular PCR-screening of asymptomatic patients and HCW; positive patients were isolated and positive HCW were excluded from work as a rule and they were only allowed to resume their work if a follow-up PCR CT-value was ≥30 and were asymptomatic or having only mild symptoms. Secondly, the use of FFP-2 masks was expanded to some long-lasting, close-contact, non-AGPs. After implementing these measures, the incidence of new cases declined gradually. Thirty-seven percent of patients died due to COVID-19.

The second outbreak (10 patients/2 HCW) was caused by the highly transmissible omicron BA.1 variant and occurred in February 2022, where transmission occurred on shared rooms despite the extra infection control measures. It was controlled much faster, and the clinical impact was low as the majority of patients was vaccinated; no patients died and symptoms were relatively mild in both patients and HCW.

## Introduction

In hospital, patients, healthcare workers (HCW) and visitors come together in close contact for long periods of time, resulting in an ideal setting for viral transmission. In the early days of the COVID-19 pandemic, testing and cohorting of COVID-19 patients on specialized COVID-19-wards, universal masking on non-COVID-19 wards and social distancing in other situations were the cornerstones of infection control policy in hospitals [1,2]. The introduction of vaccination by the end of 2020 was of high importance for reducing morbidity and mortality, but the effect on the transmission was limited due to the emergence of new variants of concern (VOC) [2].

Despite these measures, COVID-19 outbreaks in hospitals have been described frequently, also on wards with immunocompromised patients [[3], [4], [5]]. COVID-19 mortality is higher in immunocompromised patients [6] and especially in patients with haematological malignancies [7,8]. In the literature, a decrease in COVID-19 mortality was reported since the start of the pandemic. This can be explained by better understanding of the disease and specific preventive and treatment options against COVID-19, including vaccination, remdesivir, monoclonal antibodies, and convalescent plasma [9]. Furthermore, the pathogenicity of the various SARS-CoV-2 variants differs considerably, depending on their capacity to infect the lower airways [10] for evading immunity based on former variants [[10], [11], [12]] and vaccination [13].

Nowadays, molecular techniques are very helpful in outbreak management, allowing for early detection of infection and analysis of the introduction and transmission routes of SARS-CoV-2 variants. Pre-symptomatic COVID-19 positive individuals have been shown to be highly contagious with high viral loads, and the search for these patients and HCW by PCR screening is crucial to be able to interrupt the transmission in an outbreak setting [14]. Whole Genome Sequencing (WGS) techniques provide the possibility to distinguish ongoing transmission from new introductions, having the potential to guide the outbreak management team in their strategy if results become available within days [15,16].

In this article, we describe the management of two sequential COVID-19 outbreaks in the haematology-oncology department of our hospital, which is considered to have the highest level of infection control standards of all departments in our hospital. Haematology patients are at high risk for poor outcome of COVID-19 infection [[6], [7], [8]] as well as any other viral respiratory infection, and the HCW of the department were very aware of this, having experienced an outbreak of parainfluenza-3 in the summer of 2016 [17].

## Methods

### Molecular detection of SARS-CoV-2

For combined respiratory samples of nasopharynx and throat, collected during the first outbreak, RNA extraction was performed either via the chemagic Viral DNA/RNA 300 Kit H96 kit (Perkin Elmer) or via the MagNA Pure 96 DNA and Viral NA Small Volume Kit (Roche Diagnostics) according to the manufacturer's instructions, followed by elution in a 100 μL volume (50 μL elution buffer + 50 μL water). Both extraction systems yield highly comparable Cycle Treshold (CT) values and limits of detection [18].

For the combined samples collected during the second outbreak, RNA extraction was performed exclusively using the chemagic Viral DNA/RNA 300 Kit H96 kit (Perkin Elmer) according to the manufacturer's instructions followed by elution in a 100 μL volume (50 μL elution buffer + 50 μL water).

In the early days of the COVID-19 pandemic, the National Institute for Public Health and Environment composed an external quantitative quality assessment panel of predefined samples with known viral SARS-CoV-2 loads, which was sent to all Dutch microbiological laboratories for the local validation of their SARS-CoV-2 PCR-method. [19] From this, we established that a CT value of 30 and 35 equals to 3.2 and 1.7 log_10_ copies/mL respectively.

### Whole Genome Sequencing (WGS)

In November 2020, our laboratory set up a WGS pipeline for SARS-CoV-2. Whole-genome sequencing of SARS-CoV-2 was performed as previously described [20]. Bioinformatic analysis was performed using an in-house developed pipeline MACOVID (https://github.com/MUMC-MEDMIC/MACOVID) that is based on Artic v1.1.3. Consensus sequences were used to construct phylogenetic trees with ncov pipeline v11 of nextstrain with all Dutch genomes in the Global Initiative on Sharing All Influenza Data (GISAID) (7-Jun-2022) as a reference. Pangolin lineages were assigned using the Pangolin COVID-19 Lineage Assigner web application on https://pangolin.cog-uk.io/.

During the outbreak on the haematology-oncology ward in 2020, this technique was used for the first time in our hospital. In the second outbreak we had further optimized the logistics and were therefore able to report results within 5–7 days. For technical reasons, only positive samples belonging to the outbreaks with a minimum viral load corresponding a CT-value of ≤32 were eligible for WGS.

### Viral load dynamics in patients and HCW during the two outbreaks

During the outbreaks we were able to study the viral load dynamics of COVID-19 positive HCW because they were excluded from work and regularly screened and their exclusion was only terminated when their viral load was decreased sufficiently, defined as having reached a CT-value of ≥30 in a constantly increasing row of CT measuring points. Also, many COVID-19 positive patients were screened many times during their admission, up to twice per week, although this was not strictly indicated.

The CT-values were converted to log_10_ transformed viral load using a standard curve as described in von Wintersdorff *et al.*, 2022 [18]. The longitudinal data of HCWs and patients were used to compare the viral load dynamics. The first positive PCR test was set as day 0. Generalized additive models (GAM) were used to compare viral load dynamics between patient and HCW. These analyses were performed using R statistical software (version 4.2.1), GAM models were created using the MGCV (version 1.8-40) and Tidymv (version 3.3.2) packages, and the figures were made with ggplot2 (version 3.3.6).

### Description of the haematology-oncology ward

The ward is situated on the top floor of our hospital, which was opened in 1989. The ward consists of eight single patient rooms, seven 2-person rooms and four 4-person rooms, resulting in a maximum capacity of 38 patients. In 2022, the hospital wide mean duration of admission for the total patient population on the ward was 10 days, for haematology patients it was 28 days. Care on the ward is provided by 33 full time equivalent (FTE) nursing personnel (37 nurses) and approximately 6 FTE physicians.

### Normal infection control measures in the Maastricht University Medical Centre during the COVID-19 pandemic before the outbreaks occurred

In the non-COVID-19 departments of our hospital, HCW had to wear a type II-R surgical mask during patient contacts within 1.5 m distance; for AGP it was advised to change to an FFP-II mask. For other contact moments it was strongly advised to adhere to 1.5 m social distancing. In situations where this was not physically possible, it was also obliged to wear type-II-R surgical masks. In 2 and 4-person rooms, contacts between patients was reduced to a minimum; the curtains in between beds had to remain closed, patients had to eat on their bed and were advised to wear a type II-R surgical mask when not in bed.

In the haematology-oncology department there were additional infection control measures. Patients with respiratory symptoms were admitted in isolation awaiting the PCR result of various respiratory viruses including SARS-CoV-2; if the suspicion of COVID-19 was low, contact-droplet isolation in a single room was advised. If suspicion was high, patients were put in aerogenic-contact isolation. Patients were allowed to be visited by only one relative without respiratory symptoms, wearing a surgical mask during their whole visit. HCW with symptoms typical for COVID-19 were tested with a SARS-CoV-2-PCR of a combined nasopharynx-throat sample and were allowed to continue their work awaiting the result, if they had only mild symptoms and wore an FFP-2 mask during patient contact moments. If the CT-value proved <30 (high viral load), an exclusion from work was implemented. They were regularly re-tested and allowed to resume their work wearing an FFP-2 mask at all times, when their PCR CT-value became ≥30. They were regularly retested to follow the dynamics of the CT-value. If the CT-value became ≥35 they were considered to be not infectious anymore [19].

## Results

### Description of the outbreaks, interplay between the infection control team and molecular microbiology

**Figure 1:**
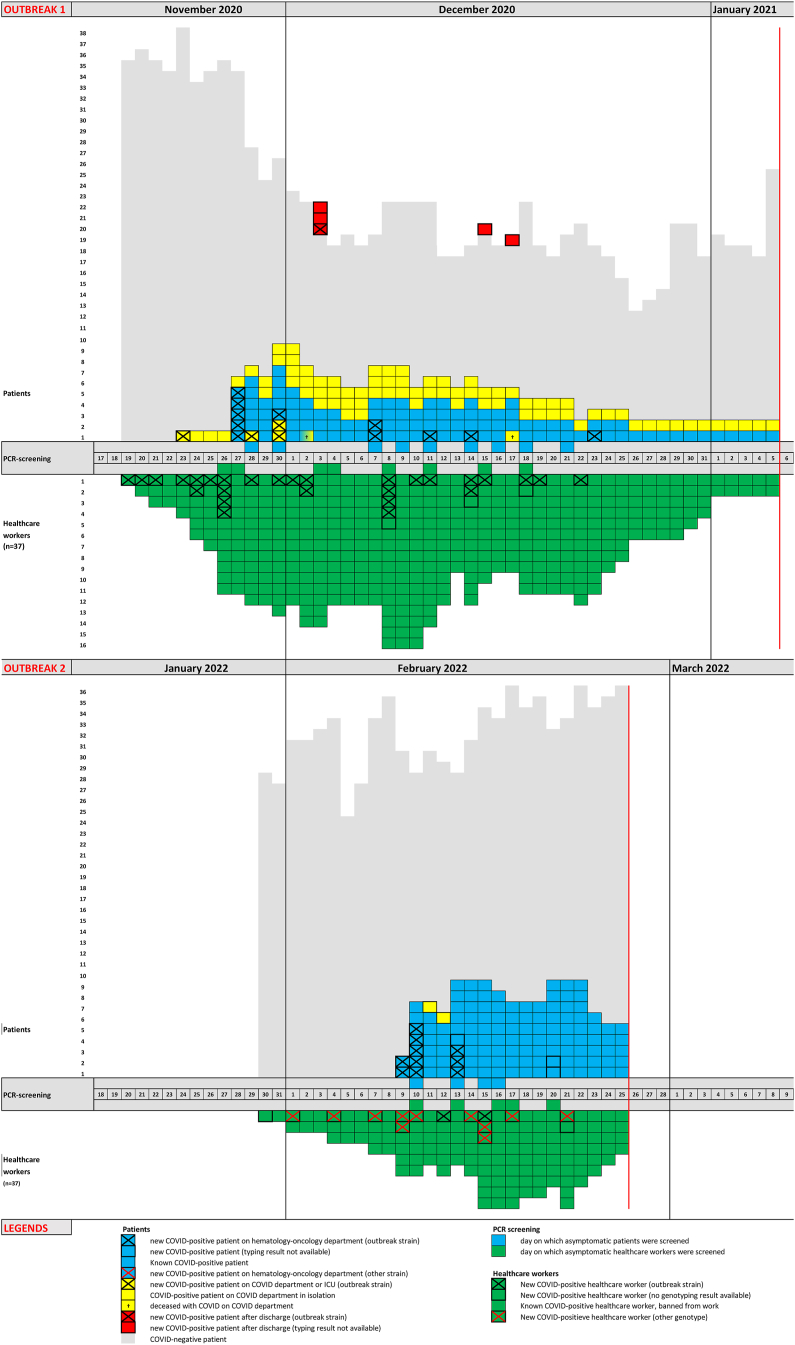
Epidemic curve 2020 and 2022.

Two COVID-19 outbreaks occurred on the haematology-oncology ward of our hospital, comprising 20 patients/31 HCW and 13 patients/14 HCW respectively, which were controlled with considerable effort. Screening of asymptomatic patients and health care workers was pivotal in this. Unfortunately, in the first outbreak, 7 patients died despite having been transferred to the Intensive Care Unit. The epidemic curves of both outbreaks are shown in Figure 1. Patient characteristics and CT-value of the first PCR test of all COVID-19-patients for both outbreaks are described in supplementary Table 1. A detailed description of the outbreaks is provided in Supplement 1. The main differences between the two outbreaks and the differences in infection control policies for patients as well as HCW is summarized in supplementary Table 2.

Molecular diagnostic results were used to optimize the infection prevention strategies. From the beginning of the outbreaks, PCR screening of asymptomatic patients was initiated in several rounds, which helped us to identify pre-symptomatic COVID-19 patients who were immediately isolated. Furthermore, in the first outbreak, PCR screening of newly admitted asymptomatic patients was performed to prevent new introductions on the ward; preferably this was done before admission, if not possible patients had to await the test result in quarantine. Isolation of positive patients was only terminated when the viral load was declining in time and the CT-value of PCR was ≥35. Also, PCR screening of asymptomatic HCW was used to combat the outbreak, and identified many positive cases. COVID-19-positive HCW were only allowed to resume their work when physically recovered and PCR CT-value ≥30. Finally, the timely availability of WGS results was very helpful for the infection control team by confirming that patients indeed belonged to a single variant outbreak and by the identification of the endpoint of an in hospital outbreak during a pandemic.

### WGS results of the outbreaks (Figure 2, Figure 3)

**Figure 2:**
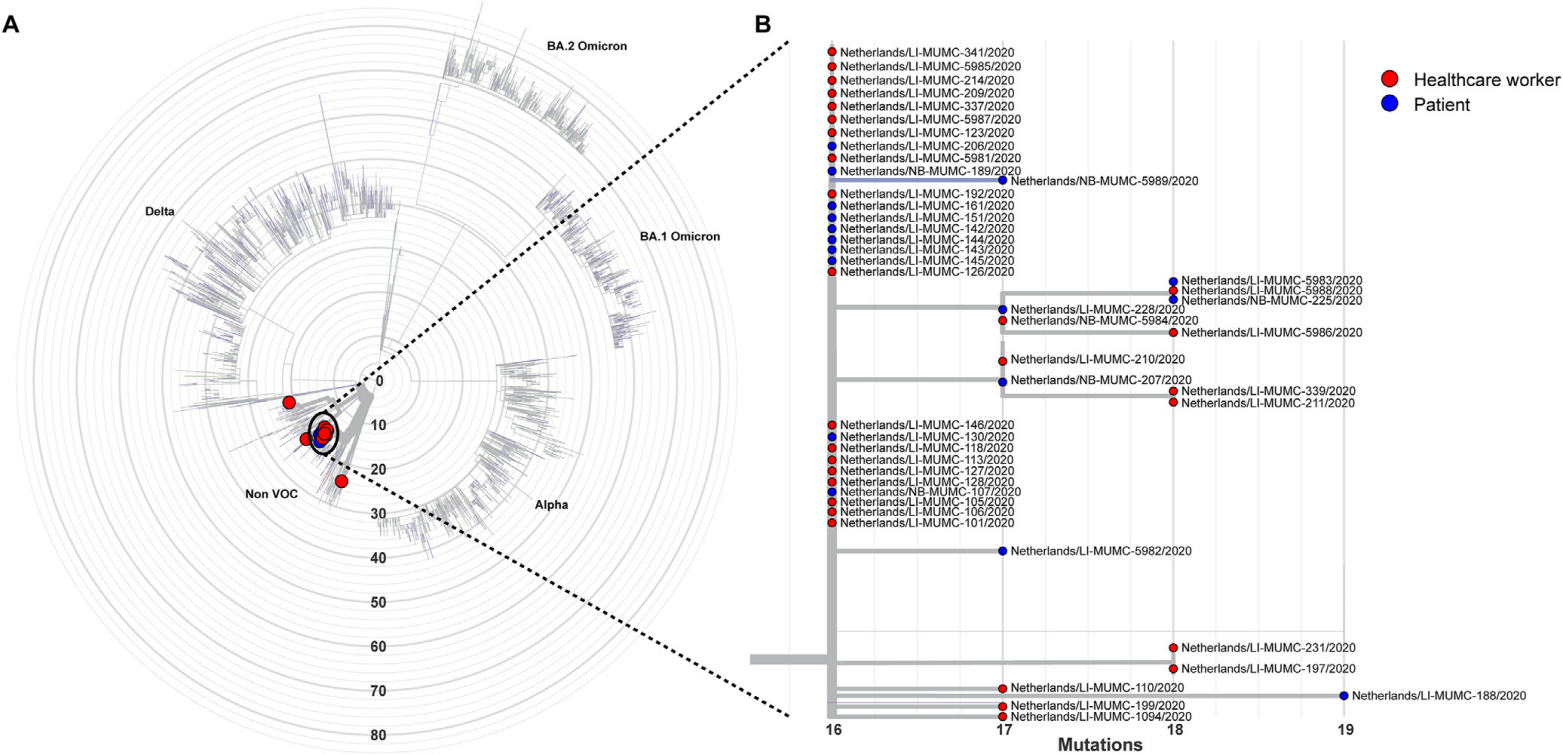
Whole Genome Sequencing results 2020. **A.** Radial phylogenetic tree of all sequenced isolates from outbreak 1. Isolates that clustered were highlighted by a black oval. **B.** Detailed phylogenetic relationship of the isolates that clustered in this study. Healthcare workers are highlighted in red, while patients were highlighted in blue. The phylogenetic tree was visualized using the auspice tool from Nextstrain (https://auspice.us/). Sample 206/2020 and sample 228/2020 belong to the same patient.

**Outbreak 1:** WGS revealed that all but three SARS-CoV-2 isolates clustered together (Figure 2). The isolates that did not cluster were obtained from two HCW who tested positive more than 2 weeks before the other subjects and one patient who tested positive on the 12th of January. The isolates that clustered together belonged to lineage B.1.221 and were characterized by the unique mutation A26513G. This genotype was compared to the GISAID international database of SARS-CoV-2 genotypes and appeared to be unique in The Netherlands and worldwide. Furthermore, it was not related to other sequenced SARS-CoV-2 isolates from within our hospital and regional public health isolates that were samples around the same period (see Figure 2).

**Figure 3:**
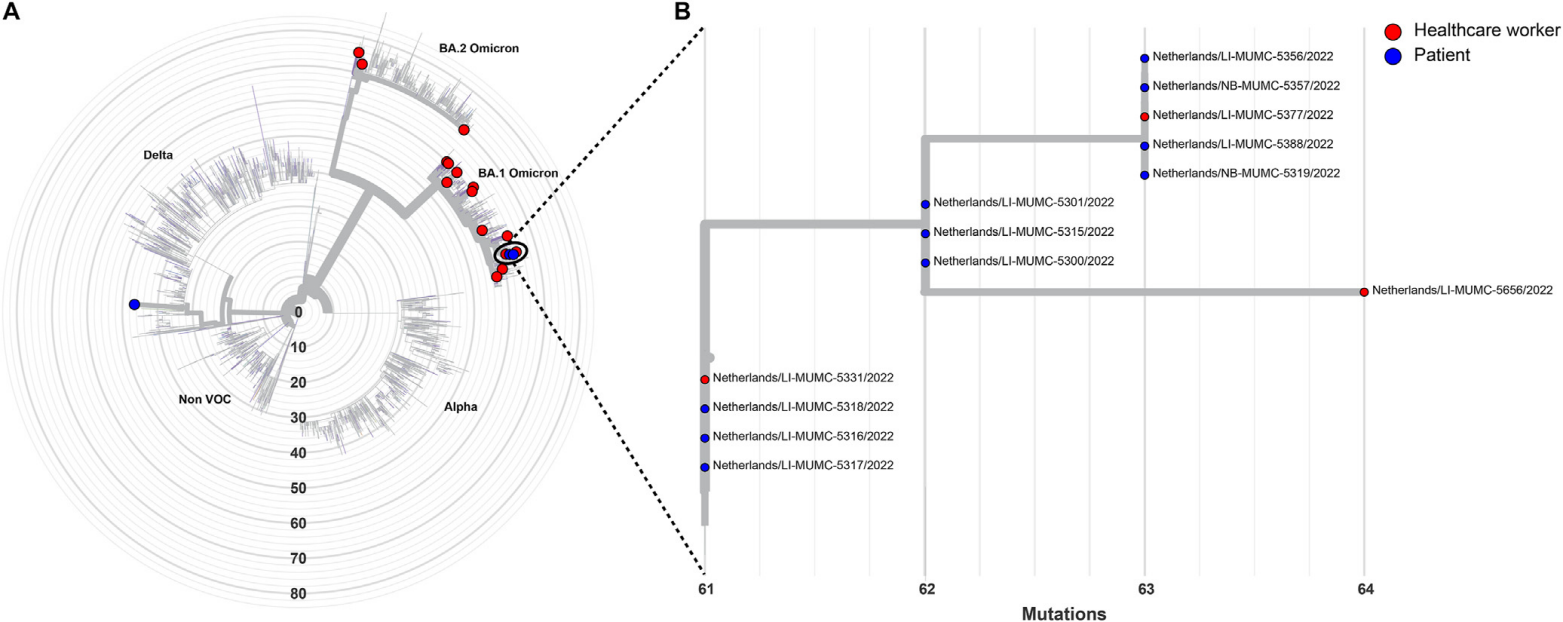
Whole Genome Sequencing results 2022. Radial phylogenetic tree of all sequenced isolates from outbreak 2. Isolates that clustered were highlighted by a black oval. **B.** Detailed phylogenetic relationship of the isolates that clustered in this study. Healthcare workers are highlighted in red, while patients were highlighted in blue. The phylogenetic tree was visualized using the auspice tool from Nextstrain (https://auspice.us/).

**Outbreak 2:** Sequencing of isolates from the second outbreak showed that only 12 SARS-CoV-2 isolates (10 patients and 3 HCW) clustered together, while 14 samples were not related to this cluster or to each other as they belonged to different sub-lineages of Omicron BA.1, BA.2 or the Delta variant (Figure 3; Table S2). The isolates that clustered together belonged to a sub-lineage of the Omicron BA.1 variant that was restricted to the hospital around the time of the outbreak.

### Patient characteristics and outcome (Supplementary Table 1)

The majority of SARS-CoV-2 infections occurred in haematology patients. In the first outbreak, seven patients were transferred to the Intensive Care Unit, but none of them survived (mortality rate 35%). In the second outbreak, symptoms were usually milder (mortality rate 8%). Information on the vaccination status of the patients in the second outbreak could not reliably be retrieved in retrospect from the patient files; the haemotologists have the impression that most of them were vaccinated but their claim could not be quantified.

### Differences between outbreaks (Supplementary Table 2)

The outbreaks occurred in sequential waves of the pandemic. The different features of the involved variants of SARS-CoV-2, in combination with advancing knowledge of the properties of the virus, caused differences in dynamics and approach.

### Viral load dynamics of patients and HCW by PCR (Supplementary Figures 1 and 2)

The viral load dynamics were compared between patient and HCW for both outbreaks, 2020 (B.1.221) and 2022 (BA.1) (Supplementary Figures 1 and 2). In both outbreaks, HCWs cleared the SARS-CoV-2 infection faster than patients, as measured by the mean time from first positive PCR-result to the first PCR-result with CT-value ≥ 35. For the outbreak in 2020: this was 16 and 49 days for HCW and patients respectively (*P*=0.002). For the outbreak in 2022 it was 52 days for HCW but with a very wide confidence interval; for patients no result could be measured. Supplementary Figure 2 suggests that patients do not readily clear the infection, but this effect is driven by 2/11 patients that were SARS-CoV-2 positive for more than 30 days.

## Discussion

In this article, we describe the course of two sequential outbreaks with two different strains of SARS-CoV-2 on the haematology-oncology ward harbouring immunocompromised patients with a very high morbidity/mortality risk. In the first outbreak, VOC alpha B.1.221, was shared by both patients and HCW, and the impact was high because vaccination was not and therapeutic options were scarcely available. In the second outbreak, VOC omicron BA1 only caused a small outbreak (with only two HCW involved); other HCW proved to be infected with genotypes that circulated outside the hospital. The clinical impact of the COVID-19 in patients was low due to the lower pathogenicity of the omicron variant and the fact that many patients were vaccinated. None of the patients needed specific COVID-19 therapy. We think that the second outbreak was smaller due to the lessons learned in the earlier outbreak, but also the fact that most patients were vaccinated may have led to a decrease of transmission [21].

In our opinion, the screening of asymptomatic patients and HCW, along with early isolation of inpatients and the exclusion from work for SARS-CoV-2 positive HCW, was very helpful in reducing the likelihood of nosocomial spread. On occasion, these individuals proved to be pre-symptomatic, with high viral loads. This explains why new COVID-19 positive patients mainly occurred in shared rooms where social distancing rules (1.5 m distance) and wearing a II-R surgical mask during transfers were not always followed. This was also true for visitors, but in these outbreaks we could not identify introductions of SARS-CoV-2 by visitors. Influencing patient and visitor behaviour is essential, but inevitably cumbersome in an outbreak situation. During the outbreaks, the virus spread over many rooms on the ward. In the second outbreak, this was linked to a rapid transmission in shared rooms for 2 or 4 patients, but in the first outbreak HCW must have played a role in transmission. An experimental study conducted by the Centres for Disease Control showed that the percentage of aerosols blocked by surgical masks is only approximately 50% during coughing and exhaling [22]. Therefore, we think that the change to wearing FFP-2 masks during procedures in which the faces of patient and HCW are close together for prolonged periods, in addition to AGP, may have been an effective intervention that helped ending this outbreak. During the first outbreak, we had to increase staff awareness of preventive measures in non-patient care moments; for example breakdowns in social distancing during meetings, breaks, but also car-pooling, and workrooms/meeting rooms too small to harbour the usual amount of people. In the second outbreak, the awareness was high and tele-conferencing had become standard.

The rapid availability of WGS results was essential in disentangling the cause and course of both outbreaks [15,16,23]. The short WGS turnaround time, combined with the epidemiological data, guided the outbreak management team regarding the necessary interventions. During the first outbreak, the WGS pipeline was under development and the turnaround time was about 10 days, which is too long for guiding infection prevention measures. Results were especially helpful in identifying a single strain outbreak shared by patients and HCW. Not every isolate was genotyped in the middle of the outbreak, due to shortage of staff ion the molecular microbiology department. Furthermore, samples with a viral load too low for sequencing, or the non-availability of specimens from patients who had been tested outside the hospital were also limitations. The absence of those results was of minor importance at that moment, because infection control measures were identical irrespective of genotype. As the incidence of new patients eventually diminished, WGS was important in declaring the outbreak to have ended. For this article, the non-genotyped strains were all retrospectively typed, and it was demonstrated that all strains indeed belonged to the outbreak. In the second outbreak, the outbreak strain circulated for a shorter time frame and mainly among patients. HCW proved to be infected by several other strains.

The viral load dynamics of the incriminated outbreak strains were very different. In general, viral loads measured by PCR of patients were higher and the decline over time was slower compared to HCW. The viral load did not correlate with the severity of symptoms; this was especially noticed in HCW in the 2020 outbreak, as symptoms caused by the omicron variant in the second outbreak were less severe. The latter is most likely due to the mitigation of symptoms due to vaccination, but the information on the vaccination status in the patient files of the second outbreak was incomplete. Due to the complexity of an outbreak situation, it was not possible to estimate the relation between viral load on the reproduction number on the ward. In our hospital, the return-to-work policy for HCW in 2020 was viral load based; only when their PCR CT-value returned to values ≥30 they were allowed to resume working in the hospital. The cut-off points of CT 30 and 35 (3.2 and 1.7 log_10_ copies/mL) were relatively arbitrarily chosen but prompted by a reasoned choice. On one hand, there was the risk of reintroduction of the virus into the hospital by recovered SARS-CoV-2 infected unvaccinated HCW with persistently high viral loads [28]. On the other hand, there was the danger of shortage of HCW caring for the hospitalized patients. This resulted in a small subset of HCW that had symptomatically recovered from COVID-19 but were not allowed to resume their work because of persistent high viral loads for many weeks. However, some HCW developed long-COVID symptoms for a long time period. During the pandemic it became clear that vaccination protected for severe disease, but vaccinated persons could still be infectious to other individuals.

The relationship of viral load and transmissibility of SARS-CoV-2 still must be elucidated. Although there are several influencing factors, such as coughing and sneezing, there is evidence that a higher viral load leads to a higher risk of transmission [24,25]. Even though there are various technical factors during the period from sampling until PCR CT-value result [26], the follow-up samples of positive HCW showed a gradual decline in viral load, but some remained high for longer time. It is now generally believed that immunocompetent people are not infectious ≥ 14 days after start of symptoms, as viral cultures from respiratory samples become negative in immunocompetent persons [27]. Therefore, our HCW policy was adapted in 2022 to a non-PCR follow-up policy, as the risk of transmission after 7 days of isolation was considered low [25]. Nowadays, for immunocompromised haematological and solid transplant patients, the infectious period is generally estimated to be 14–21 days [[29], [30], [31]], but as we also have experienced, it is sometimes much longer [32].

## Conclusions

Our in-hospital COVID-19 outbreaks were very challenging for our infection control unit, particularly during the beginning of the pandemic when the circulating SARS-CoV-2 variant was of high virulence, therapeutic options were not available, and the outbreak occurred on a ward consisting of immunocompromised patients. Guidelines to prevent transmission were based on advancing but incomplete understanding of the virus dynamics, combined with the fact that adherence to enacted infection control guidelines was sometimes unintentionally suboptimal. Furthermore, behaviour of patients and visitors was regularly difficult to manage. In our hospital, frequent screening of asymptomatic patients and HCW in outbreak situations proved essential as many new cases with often high viral loads were identified. These individuals were considered the main drivers of ongoing transmission. As a consequence of screening, we detected cases in a very early phase with low viral loads, which were possibly not yet infectious to others, but we learned that most of them progressed to high viral loads within a few days, so they were also isolated or excluded from work pending the results of follow-up testing. The introduction of WGS for SARS-CoV-2 and the further optimization of the turn-around time to results to 5–7 days gave insight into viral dynamics, and was an effective instrument to identify outbreaks in an early phase and declare outbreaks to have ended with high certainty.

## Author contributions

WC van der Zwet: Conceptualization, methodology, validation, investigation, visualization, supervizion, project administration, writing. EA Klomp-Berens: Conceptualization; methodology, validation, investigation, visualization, reviewing and editing. AMP Demandt: Conceptualization, investigation, visualization, reviewing and editing. J Dingemans: Validation, formal analysis, visualization, reviewing and editing. BMJW van der Veer: Validation, formal analysis, visualization, reviewing and editing. LB van Alphen: Validation, formal analysis, reviewing and editing. JACM Dirks: Validation, investigation, reviewing and editing. PHM Savelkoul: Validation, resources, reviewing and editing.

## Conflicts of interest

None to declare.

## Funding source

None to declare.

## Ethics statement

This article is a retrospective description and analysis of an hospital outbreak, which is an essential element of quality improvement of infection prevention as a part of patient care in general. Ethical approval was not deemed necessary.

## Appendix A Supplementary data

Multimedia component 1 Multimedia component 1

Multimedia component 2 Supplementary Figure 1: Follow-up viral loads patients and HCW 2020. Viral load dynamics of patients and healthcare workers of the 2020 outbreak. SARS-CoV-2 negative samples are −1 log10 and day 0 represent the first SARS-CoV-2 positive PCR test. The dashed horizontal red lines represent Ct-values 30 (3.2 log10 copies/mL) and 35 (1.7 log10 copies/mL). Multimedia component 2

Multimedia component 3 Supplementary Figure 2: Follow-up viral loads patients and HCW 2020. Viral load dynamics of patients and healthcare workers of the 2022 outbreak. SARS-CoV-2 negative samples are −1 log10 and day 0 represent the first SARS-CoV-2 positive PCR test. The dashed horizontal red lines represent Ct-values 30 (3.2 log10 copies/mL) and 35 (1.7 log10 copies/mL). Multimedia component 3
