# Supplementary material for: Analysis of two sequential SARS-CoV-2 outbreaks on a haematology-oncology ward and the role of infection prevention
Source: Infect Prev Pract. 2024 Jan 6;6(1):100335. doi: 10.1016/j.infpip.2023.100335 (PMC10826166; doi:10.1016/j.infpip.2023.100335)
Supplement: Multimedia component 1 [file mmc1.docx]

SUPPLEMENT 1 EXTENDED DESCRIPTION OF THE OUTBREAKS AND INFECTION CONTROL MEASURES

**Description Outbreak 1, November-December 2020**

In the second half of November 2020, seven members of the nursing staff of the department became symptomatic and tested positive for COVID-19 (PCR CT 11-19). In addition, a patient that was discharged from the ward five days before was readmitted with fever and diarrhoea, and tested COVID-19 positive. Transmission of SARS-COV-2 on the ward was suspected. Therefore, all admitted patients, that were asymptomatic at that moment, were investigated by PCR-screening and another five appeared positive. Furthermore, screening of asymptomatic HCW identified four positive persons. An outbreak-management-team (OMT) was convened, and on November 29th the following measures were installed: (1) The haematology-oncology ward was temporarily closed to new admissions; admissions that could not be postponed, were realized on other wards. For patients who were scheduled for chemotherapy or stem cell transplantation, the help of neighbouring hospitals was organized; (2) The admitted asymptomatic patients and all asymptomatic regular HCW were prospectively screened by PCR (patients thrice a week, and HCW twice a week); (3) For visitors, the maximum of one visitor per patient per day and two for the whole admission period was maintained. For the 2- and 4-person rooms, a scheme was developed to ensure that a maximum of one visitor per room was present at a time; (4) Because of the lack of available single rooms, COVID-19 positive patients were co-horted; (5) Whenever their medical situation allowed it, COVID-19 positive patients were transferred to the specialized COVID-19 ward.

Despite our bundle of infection control measures, new positive patients (from which five became positive after discharge) as well as HCWs were identified. On December 7th, the positive patients were co-horted at one side of the ward and the other side was reopened for new admissions. Additional measures were constituted: (1) For the prevention of new viral introductions on the ward, a standard SARS-COV-2 screening 24-48 h before admission for all new electively admitted patients was constituted; (2) Nursing personnel had to change their type-II surgical mask every hour, to prevent saturation and decrease of its protective function: (3) All non-patient related activities (formal and informal meetings, changing clothes in dressing rooms, etc.) of HCW on the ward were critically appraised for the risk of transmission and improved if possible; (4) HCW were required to wear a FFP-2 mask in three strictly defined non-AGP procedures, in which the face of the HCW becomes close to that of a asymptomatic non-COVID-19 patient for a longer period, namely the intensive care of the mouth, the care for tunnelled and non-tunnelled central venous jugular catheters and nebulization of medications; (5) For the non-COVID-19 cohort, the use of 2- and 4-person rooms was avoided as much as possible; (6) Furthermore, an addition to the normal HCW test-policy was installed. It became clear that the majority of the newly identified asymptomatic/pre-symptomatic COVID-19 patients and HCW had a low CT-value (15-20) but a few had a high CT-value (>30) that rapidly decreased to a very low CT-value on retesting a few days later (very early detection). Therefore, HCW that tested positive for the first time with a high CT-value were also banned from work pending the result of retesting 48 hours later.

The total outbreak on the ward eventually comprised 20 patients and 31 HCW. As there was no effective treatment at that time and the circulating Wuhan strain had a high pathogenic potential, the morbidity and mortality were high (Table 1). An important percentage of COVID-19 positive HCW had long-lasting complaints of the infection and remained incapacitated far longer than the period that their CT-value required them to be excluded from work. Therefore, extra personnel had to be recruited. On the other hand, there were HCW who were physically recovered but remained excluded from work due to remaining high viral loads (CT-value < 30).

As new nosocomial cases occurred less frequently during December, the regular high-frequency screening was stopped on December 21st. The last positive symptomatic patient was identified on December 23rd. On January 5th the outbreak was officially declared to have ended, since new cases were not identified for the maximum incubation period of COVID-19 (11 days). In the following week another symptomatic patient and HCW who had cared for this patient tested positive, but WGS showed their strains were different from each other and not related to the outbreak strain.

**Description Outbreak 2, February 2022**

On February 9^th^, two symptomatic patients and two symptomatic HCW tested positive for COVID-19. In the week before, three other HCW had also become symptomatic and COVID-19 positive. The next morning, another two symptomatic patients tested positive. Therefore, it was decided to screen all remaining admitted asymptomatic patients. This screening detected another three positive patients, and one new positive HCW who was not on duty that day. We decided to continue the screening of asymptomatic patients. At first, HCW were only tested when symptomatic, but as new symptomatic cases occurred soon after it was decided on February 16^th^ to also screen asymptomatic HCW. All infection control measures were the same as in the first outbreak. The visitor’s policy, that had been mitigated in the context of the upcoming less pathogenic omicron variant of COVID-19, was again tightened: only one visitor per day was allowed instead of two separate visitors per day. Furthermore, on shared rooms a scheme was re-introduced preventing the presence of multiple visitors at the same time. Positive HCW were excluded from work with a CT-value <30 and were allowed to resume their work under the following conditions: (1) asymptomatic after 14 days, irrespective of CT-value; (2) still symptomatic after 14 days but a CT-value ≥30 on retesting, or (3) a CT-value <30 but >24 hours asymptomatic. On screening, new positive asymptomatic patient cases were found on February 13^th^ and 20^th^. The CT-values of the new patients on February 20^th^ were 34 and 35 respectively, which implicated their viral load was too low to get a WGS result. Two days later they both tested negative. The outbreak was declared to have ended on February 26^th^.
