# Supplementary figures and images for: Analysis of two sequential SARS-CoV-2 outbreaks on a haematology-oncology ward and the role of infection prevention
Source: Infect Prev Pract. 2024 Jan 6;6(1):100335. doi: 10.1016/j.infpip.2023.100335 (PMC10826166; doi:10.1016/j.infpip.2023.100335)

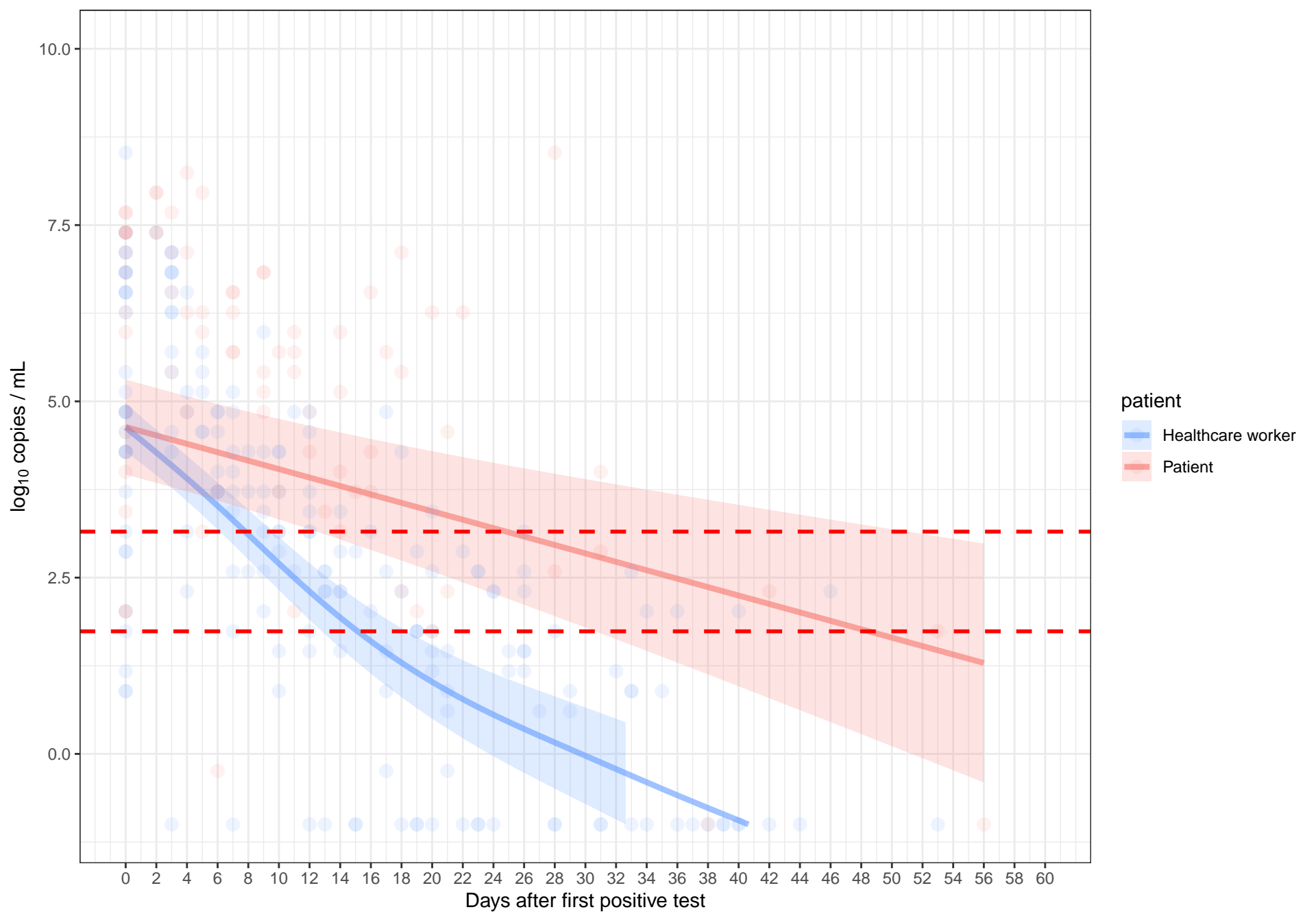

Supplement: Multimedia component 2 — Supplementary Figure 1: Follow-up viral loads patients and HCW 2020. Viral load dynamics of patients and healthcare workers of the 2020 outbreak. SARS-CoV-2 negative samples are −1 log10 and day 0 represent the first SARS-CoV-2 positive PCR test. The dashed horizontal red lines represent Ct-values 30 (3.2 log10 copies/mL) and 35 (1.7 log10 copies/mL). [file mmc2.pdf]

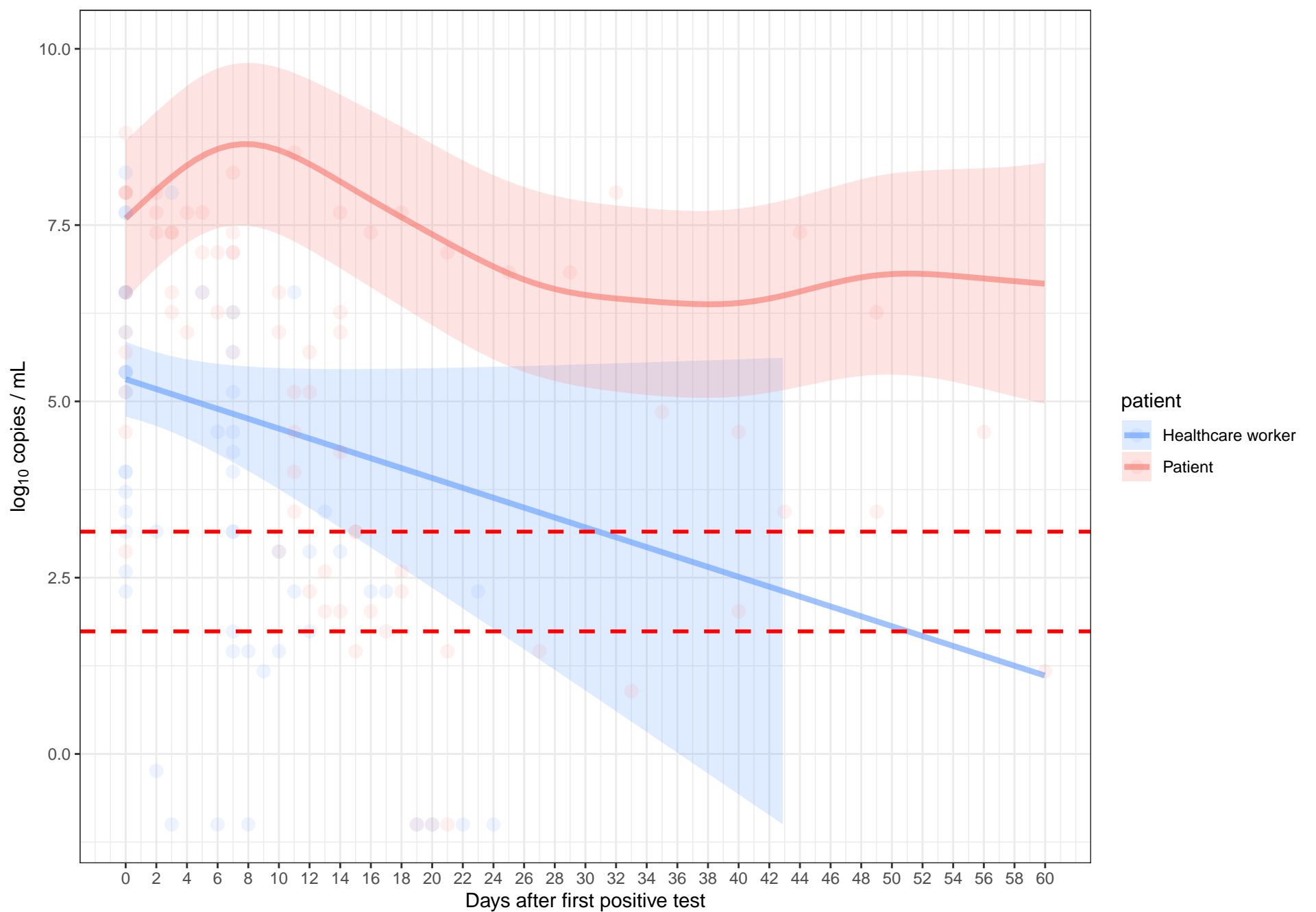

Supplement: Multimedia component 3 — Supplementary Figure 2: Follow-up viral loads patients and HCW 2020. Viral load dynamics of patients and healthcare workers of the 2022 outbreak. SARS-CoV-2 negative samples are −1 log10 and day 0 represent the first SARS-CoV-2 positive PCR test. The dashed horizontal red lines represent Ct-values 30 (3.2 log10 copies/mL) and 35 (1.7 log10 copies/mL). [file mmc3.pdf]
