# Supplementary material for: Analysis of two sequential SARS-CoV-2 outbreaks on a haematology-oncology ward and the role of infection prevention
Source: Infect Prev Pract. 2024 Jan 6;6(1):100335. doi: 10.1016/j.infpip.2023.100335 (PMC10826166; doi:10.1016/j.infpip.2023.100335)
Supplement: Multimedia component 4 [file mmc4.docx]

**Table 1 Patient characteristics**

**Outbreak 2020**

| **No** | **Age** | **Sex** | **Specialism** | **Disease and treatment** | **Date of**  **positive PCR** | **Admission day on ward** | **CT** | **SARS-CoV-2 genotype** | **COVID-19 symptoms** | **COVID-19 antibodies** | **Treatment** | **Deceased** | **Due to COVID?** |
| --- | --- | --- | --- | --- | --- | --- | --- | --- | --- | --- | --- | --- | --- |
| 01 * | 69 | F | Hematology | Multiple myeloma  Autologous Tx | 23.11.20 | Outpatient | 16 | Outbreak | No (symptoms started 29.11) | Not tested | Dexamethason, ICU admission | Yes | Yes |
| 02 | 43 | M | Hematology | Refractory Hodgkin Lymphoma  Autologous Tx | 27.11.20 | 17 | 22 | Outbreak | Yes (fever and cough | Not tested | No | No | N.a. |
| 03 | 77 | F | Oncology | Metastatic stomach cancer  Chemotherapy | 27.11.20 | 12 | 14 | Outbreak | Loss of taste and smell | Not tested | No | No | N.a |
| 04 | 71 | F | Oncology | Metastatic breast cancer  Diagnostic procedure | 27.11.20 | 14 | 15 | Outbreak | Oxygen therapy | Not tested | Dexamethason | No | N.a. |
| 05 | 79 | M | Oncology | Metastatic colorectal cancer  Chemotherapy | 27.11.20 | 11 | 15 | Outbreak | Fever | Not tested | No | No | N.a. |
| 06 * | 48 | F | Oncology | Metastatic breast cancer  Chemotherapy | 27.11.20 | 7 | 15 | Outbreak | Fever and Oxygen therapy | Not tested | Dexamethason, ICU admission | Yes | Yes |
| 07 | 66 | M | Hematology | Aggressive B-cell NHL  Chemotherapy | 28.11.20 | Outpatient | 20 | Outbreak | Fever and Oxygen therapy | Not tested | Dexamethason | No | N.a. |
| 08 | 52 | F | Oncology | Metastatic rhabdomyosarcoma  CTX | 30.11.20 | Outpatient | 17 | Outbreak | Cough, fever and Oxygen therapy | Not tested | Dexamethason | No | N.a. |
| 09 | 63 | M | Hematology | Acute myeloid leukemia  Allogenic Tx  Immune suppression because of Graft-versus-host-disease | 30.11.20 | 6 | 29 | Outbreak | Fever and later on dyspnea | Not tested | Dexamethason | Yes | No |
| 10 | 54 | M | Hematology | Multiple myeloma  Autologous Tx | 30.11.20 | Outpatient | 17 | Outbreak | Fever and dyspnea | Not tested | Dexamethason | No | N.a. |
| 11 | 37 | M | Hematology | Acute lymphoblastic leukemia  Intensive CTX | 03.12.20 | Outpatient | N.a. | N.a. | No | Not tested | No | No | N.a. |
| 12 | 64 | M | Oncology | Metastatic esophageal cancer  Best supportive care | 03.12.20 | Outpatient | 15 | Outbreak | Dyspnea | Not tested | No | Yes | Possibly contributed (also progressive disease) |
| 13 | 62 | M | Hematology | Acute myeloid leukemia  Between intensive CTX and allogeneic Tx | 03.12.20 | Outpatient | N.a | N.a | No | Not tested | No | No | N.a. |
| 14 * | 71 | M | Hematology | Aggressive B-cell NHL  Autologous Tx | 07.12.20 | 41 | 14 | Outbreak | Cough and dyspnea after 3 weeks | 04.01.21 Negative  07.01.21 Positive | ICU admission, Dexamethason, Remdesivir, convalescent plasma | Yes | Yes |
| 15 * | 54 | M | Hematology | Hairy cell leukemia  Chemotherapy | 08.12.20 | 17 | 26 | Outbreak | Cough and later on dyspnea and O2-therapy | 01.01.21 Negative 07.01.21 Positive | ICU admission, Dexamethason, Remdesivir, convalescent plasma | Yes | Possibly contributed (died because of cardiac failure) |
| 16 | 53 | F | Hematology | Relapse Hodgkin lymphoma  Autologous Tx | 11.12.20 | 19 | 24 | Outbreak | No but 16.12 fever | Not tested | No | No | N.a. |
| 17 * | 57 | F | Hematology | Acute myeloid leukemia  AllogeneicTx | 14.12.20 | 27 | 25 | Outbreak | No but 16.12 fever | 08.01.21 Negative | ICU admission, Dexamethason, Remdesivir, convalescent plasma | Yes | Possibly contributed but also relapse of leukemia and abstinence of therapy |
| 18 | 23 | F | Hematology | Acute lymphoblastic leukemia | 16.12.20 | Outpatient | N.a. | N.a. | Cough | Not tested | No | No | N.a. |
| 19 * | 59 | M | Hematology | Primary central nervous system lymphoma  Autologous Tx | 23.12.20 | 8 | 34 | Outbreak | No but 01.01.21 fever | 15.01.21 Negative 18.01 positive | ICU admission, Dexamethason, Remdesivir, convalescent plasma | Yes | Yes |
| 20 * | 57 | M | Hematology | T-lymphoblastic lymphoma  Allogeneic Tx | 07.01.21 | 31 | 23 | Not related | Cough and fever | 20.01.21 positive | ICU admission, Dexamethason, Remdesivir | Yes | Yes |

N.a.; not avalailable/applicable;. CTX; chemotherapy, Tx;transplant; NHL; Non-Hodgkin lymphoma;

* ICU admissionO**utbreak 2022**

| **No** | **Age** | **Sex** | **Specialism** | **Disease and treatment** | **Date of**  **positive**  **PCR** | **Admission**  **day on ward** | **CT** | **SARS-CoV-2**  **genotype** | **COVID-19 symptoms** | **COVID-19 antibodies** | **COVID-19**  **treatment** | **Deceased** | **Due to COVID?** |
| --- | --- | --- | --- | --- | --- | --- | --- | --- | --- | --- | --- | --- | --- |
| 01 | 61 | M | Oncology | Colorectal cancer  Adj chemotherapy | 09.02.22 | 22 | 25 | Outbreak | Cough, fever  (on 10.02) | Positive | No | No | N.a. |
| 02 | 63 | F | Internal medicine | Dementia | 09.02.22 | 9 | 23 | Outbreak | No | N.a. | No | No | N.a. |
| 03 | 30 | M | Hematology | Acute myeloid leukemia  Intensive CTX | 10.02.22 | 22 | 18 | Outbreak | No | Positive | No | No | N.a. |
| 04 | 58 | M | Hematology | Acute lymphoblastic leukemia  Intensive CTX | 10.02.22 | 24 | 18 | Outbreak | No | Positive | No | No | N.a. |
| 05 | 53 | F | Hematology | Acute myeloid leukemia  Intensive CTX | 10.02.22 | 23 | 13 | Outbreak | No | Positive | Sotrovimab | No | N.a. |
| 06 | 42 | F | Hematology | Aggressive B-cel NHL  Autologous Tx | 10.02.22 | 3 | 21 | Outbreak | No | Positive | No | No | N.a. |
| 07 | 59 | F | Hematology | B-cel NHL  Autologous Tx | 10.02.22 | 9 | 13 | Outbreak | Cough | Positive | No | No | N.a. |
| 08 | 61 | F | Hematology | Aggressive B-cel NHL  Chemotherapy | 13.02.22 | 18 | 13 | Outbreak | No | Positive | Sotrovimab | No | N.a. |
| 09 | 50 | M | Hematology | B-cel NHL  Allogeneic Tx | 13.02.22 | 6 | 31 | Outbreak | No | Positive | Sotrovimab | No | N.a. |
| 10 | 57 | F | Hematology | Aggressive B-cel NHL  Autologous Tx | 13.02.22 | 4 | 39 | N.a. | No | Positive | No | No | N.a. |
| 11 | 64 | M | Hematology | Acute myeloid leukemia  Allogeneic Tx | 13.02.22 | 25 | 10 | Outbreak | Fever (non-neutropenic) | Positive | No | No | N.a. |
| 12 | 66 | F | Hematology | Myelofibrosis  Allogeneic Tx | 20.02.22 | 6 | 35 | N.a. | No | Positive | Sotrovimab | Yes | No |
| 13 | 66 | F | Hematology | T-cel NHL  Autologous Tx | 20.02.22 | 4 | 34 | N.a. | No | Positive | Sotrovimab | No | N.a. |

*Adj; adjuvant. CTX; chemotherapy, Tx;transplant; NHL; Non-Hodgkin lymphoma; N.a.; not available/applicable

*Information on the vacccination status of the patients was not reliably recorded in the patient files
