# Supplementary material for: Analysis of two sequential SARS-CoV-2 outbreaks on a haematology-oncology ward and the role of infection prevention
Source: Infect Prev Pract. 2024 Jan 6;6(1):100335. doi: 10.1016/j.infpip.2023.100335 (PMC10826166; doi:10.1016/j.infpip.2023.100335)
Supplement: Multimedia component 5 [file mmc5.docx]

**Table 2 Differences between outbreaks**

|  |  | **Outbreak 1** | **Outbreak 2** |
| --- | --- | --- | --- |
| **Period** |  | Nov 2020- Jan 2021 | Feb 2022 |
| **SARS-CoV-2 variant** |  | B.1.221 (alpha) | B.A.1 (omicron) |
| **Duration** |  | 38 | 17 |
| **Number of screening rounds asymptomatic individuals** |  | Patients 9, HCW 6 | Patients 3, HCW 4 |
| **Patients** | Total | 20 | 13 |
|  | With outbreak strain | 16 (80%) | 10 (77%) |
|  | Deceased | 7 | 0 |
|  | IP-policy haematologic patients | **Start Isolation:**   - Symptomatic and PCR positive - Asymptomatic and PCR positive   **End of isolation:**  >=21 days after start symptoms AND 72 h free of COVID symptoms AND 2 PCR’s negative | **Start Isolation:**   - Symptomatic and PCR positive - Asymptomatic and PCR positive   **End of isolation:**   - >=14 days after start symptoms AND >=24 h free of symptoms - Still symptomatic AND clinical improvement. Consider PCR; if CT >=35 stop isolation |
|  | Vaccination | Not available | Available ^1)^ |
|  | Therapeutic antiviral options | Remdesivir and convalescent plasma became an option during the outbreak but when patients were already admitted to the ICU | Sotrovimab |
| **Health care workers** | Total | 31 | 15 |
|  | With outbreak strain | 28 (90%) | 2 (13%) |
|  | IP policy | **Ban from work:**   - symptomatic with CT<30, - asymptomatic with any CT-value (follow up CT-dynamics after 48 h)   **Return to work:**   - Follow-up CT-value with interval of several days (depending on expectation of CT-value becoming >=30: weekly if CT >=25, every 3 days if CT 25-29. - asymptomatic or mild symptoms AND CT >=30 | **Ban from work:**   - symptomatic with CT<30, - asymptomatic with any CT-value (follow up CT-dynamics after 48 h)   **Return to work:**   - asymptomatic and 7 days after start symptoms |
|  | Vaccination | Not available | Available ^2)^ |

1. The vaccination status of patients was not reliably recorded in the patient files. In retrospect, the heamatologists have te impression that most of the patients were vaccinated, but their claim cannot be quantified
2. The vaccination status of HCW is unknown as they were vaccinated voluntarily and information on vaccination was under strict privacy rules
